# Warming up for a better fever: a randomized pilot study in pediatric oncology

**DOI:** 10.1186/s40814-022-01144-7

**Published:** 2022-08-16

**Authors:** Hanno S. Krafft, Christa K. Raak, Ekkehart Jenetzky, Tycho J. Zuzak, Alfred Längler, David D. Martin

**Affiliations:** 1 Faculty of Health/School of Medicine, Witten/Herdecke University, Alfred-Herrhausen-Straße 50, 58448 Witten, Germany; 2 Department of Child and Adolescent Psychiatry and Psychotherapy, University Medical Center of the Johannes-Gutenberg-University, Mainz, Germany; 3 Department of Pediatrics, Gemeinschaftskrankenhaus, Herdecke, Germany; 4 Faculty of Medicine, University of Duisburg-Essen, Essen, Germany; 5 Department of Pediatrics, Eberhard-Karls University Tübingen, Tübingen, Germany

**Keywords:** Fever, Heat application, Antipyretics, Pediatric oncology, Pilot study, Study protocol

## Abstract

**Background:**

Fever in children is a major problem in pediatric oncology. Usual management leads to immediate antibiotic and antipyretic therapy, although there is consensus that antipyretic therapy should not be utilized with the sole aim of reducing body temperature. Increased body temperature during fever appears to be an effective modifier in terms of viral replication and enhanced host defense mechanisms against pathogens. Therefore, it might be beneficial to support febrile patients by applying gentle heat during the onset of fever to help the body to reach its new thermoregulatory set point.

**Methods:**

A randomized pilot study over 6 months will be conducted in a pediatric oncology department in an academic hospital in Germany. This study is a preparation for a multicenter clinical trial with two parallel groups concerning the efficacy of heat application vs. treatment as usual. One of the inclusion criteria is body temperatures ≥ 38.0 °C in *n* = 24 cases of patients receiving chemotherapy aged 18 months to 17 years. The first intervention consists of gentle heat application with hot water bottles at any sign of illness and onset of fever. The aim is to achieve a warm periphery equilibrated to trunk temperature of less than 0.5 °C. The second intervention is the avoidance of antipyretics. The control group receives the standard antipyretic treatment from the participating hospital. The purposes of this pilot study are proof of principle of intervention, evaluation of safety, feasibility, definition of endpoints, and to receive basic data for sample size calculation and needed resources.

**Discussion:**

The main goal is to improve the care of children with cancer by providing the best possible support for febrile episodes. If fever support by heat reduces discomfort, administration of antipyretics and maybe even antibiotics, this would be an advancement in oncological fever management. This pilot study is intended to provide a basis for a main, multicenter, randomized trial and demonstrate the practicability of heat application in febrile patients in pediatric oncology.

**Trial registration:**

German Clinical Trials Register (DRKS), DRKS00028273. Registered on 14 April 2022

**Supplementary Information:**

The online version contains supplementary material available at 10.1186/s40814-022-01144-7.

## Introduction

### Background and rationale {6a}

#### Benefits of fever

The increase in temperature during fever is a potent biological response modifier with myriad effects on elements of the immune response [1]. Fever appears to improve outcomes regarding viral replication and host defense mechanisms against pathogens [2]. Studies point out the potentially harmful effects of suppressing fever in mammals and humans [3–6]. The protective effects of fever against invading organisms results from a variable combination of direct thermic effects [7–9] and humoral [10] and cellular [11] defense enhancement. There are numerous clinical studies in humans supporting the benefits of fever: a large cohort study showed that elevated peak temperature in the first 24 h in the intensive care unit was associated with decreased mortality in critically ill patients with an infection. In patients without infection, the mortality risk progressively increased above temperatures above 39.0 °C [12]. A systematic review and meta-analysis of the effect of antipyretic medications on mortality in critically ill patients with infection concluded that the studies to date are insufficient to allow a robust estimate of the effect of pharmacological antipyresis on mortality in critically ill patients with suspected infection [13].

#### Fever management

By now, it is the consensus of numerous experts that the actual goal of antipyretic therapy is not to normalize core body temperature but to reduce discomfort in children with fever who seem distressed [14–16]. This consensus is reaching the intensive wards too [17, 18]. The usual treatment of fever is in most settings antipyresis from the very first moment, pharmacologically as well as by physical cooling [19]. Many hospitals advocate the regular use of the antipyretics paracetamol (acetaminophen), metamizol, and ibuprofen for body temperatures above a defined level (≥ 38.5 °C), although in clinical guidelines to date there is no uniform threshold for the administration of antipyretics [20]. In critically ill children with fever, a permissive temperature threshold was associated with a modest increase in the mean maximum temperature, while length of stay, duration of organ support, and mortality were similar between the groups and without serious adverse events [21].

#### Supporting the heat flow

The development and heat flow of fever is characterized by three dynamic phases. The first phase is the one of temperature rises, when the body decreases heat loss through centralization and increases heat production through shivering [22]. From the literature, we conclude that it is advantageous to support each fever phase with physical measures [23]. A recent scoping review showed that applying heat at or above body temperature by the use of electric warming blankets, hot packs, hot water bottles, or hot water foot baths is regularly used for febrile patients in some cultural spaces [24]. This application of heat might be reasonable until the body’s core temperature has reached the higher thermoregulatory set point, and heat production and heat loss are balanced in the second phase of temperature stabilization. At this point, no more heat is necessary, and the body will remain in this stadium until the third phase of falling temperature occurs. We therefore hypothesize, that in the phase of centralization, it might be helpful to bring the body to the new thermoregulatory set point more quickly and without the usual side effects by applying external heat.

#### Fever in children with cancer

Children with cancer under chemotherapy are particularly prone to febrile infections and at risk of therapy-related morbidity and mortality. Cancer is the leading cause of disease-related death in children in developed countries [25]. Infectious complications are the major obstacle to survival for children with cancer [26], and most dying children are admitted with a diagnosis of sepsis [27]. Under chemotherapy, children have low levels of immune cells so the abovementioned thermic, humoral, and cellular effects of fever may be relevant to the course of the infection. Furthermore, children with cancer are one of the only patient groups that present to the hospital every time they have a body temperature ≥ 38.5 °C, although there is evidence that a higher temperature threshold is safe in children with neutropenia undergoing chemotherapy [28]. The reason for choosing children as the patient population lies in the physiological specificity with regard to fever, as adults do not have the ability to fever to the same extent [29, 30]. They also have different preconditions and prognoses with regard to the underlying oncological disease.

#### Need for a study

We are conducting the study on children with cancer with the intention to improve their treatment and care. Also, we want them to feel more comfortable and reduce suffering from symptoms of centralization (chills, shivering, etc.). There are many publications on managing infections in children with cancer, but no studies on fever support in children with cancer. The objectives of this randomized pilot study are therefore to review feasibility and resources, provide a case number estimate, and ultimately define the potential outcome parameters and interventions of fever support by heat application for the main trial. This study protocol adheres to the SPIRIT reporting standard. The SPIRIT checklist is provided as a supplement.

### Objectives {7}

According to the feasibility and pilot study framework by Eldridge et al., this study is classified as a randomized pilot study [31]. The aims of this pilot study are defined as follows:

- Test of study design feasibility
- Optimization of intervention
- Evaluation of safety and termination criteria
- Evaluation and definition of primary and secondary outcomes for the main trial
- Determination of required sample size
- Definition of recruitment time
- Determination of relevant covariates
- Determination of resource and cost requirements

### Trial design {8}

2:1 randomized pilot study over 6 months in preparation of a multicenter clinical trial (RCT) with two parallel groups concerning the efficacy of a heat application vs. treatment as usual. Randomization was chosen, even though not strictly necessary, in order to acquire a clearer idea of the effect size.

## Methods: participants, interventions and outcomes

### Study setting {9}

For this randomized pilot study, the *Gemeinschaftskrankenhaus Herdecke*, Germany, was selected as the study site. The hospital is a pediatric oncology center in accordance with the quality criteria of the joint federal committee in Germany (G-BA). The study site is also a member of all major study groups of the medical society for pediatrics, oncology, and hematology (GPOH).

### Eligibility criteria {10}

The following are the inclusion criteria:

- Presence of oncologic disease
- Initiation of chemotherapy
- Need for hospitalization due to a core body temperature ≥ 38.0 °C
- Patients between the ages of 18 months and 17 years
- Informed consent from legal guardians and own from patients 14 years of age or older

The following are the exclusion criteria:

- Acute brain injury
- Malignant hyperthermia (ever occurred)
- Neuroleptic malignant syndrome (ever occurred)
- Hepatic cirrhosis (ever occurred)
- Acute hepatic failure
- History of stroke, seizure, or traumatic brain injury
- Intellectual disability

### Interventions

#### Explanation for the choice of comparators {6b}

Heat application and no antipyretics were chosen as an intervention to support the physiological development and thermoregulation of fever. The external heat helps the body to rapidly achieve the second phase of temperature stabilization where heat production and heat loss are balanced. Standard care with or without antipyresis was chosen as control because it represents everyday clinical practice on oncological wards and probably limits the effectiveness of fever.

#### Intervention description {11a}

The first intervention is gentle heat application in patients with cold extremities during the first hours of admission due to fever: patients present directly to the oncology ward if they have a fever ≥ 38.0 °C at home. Upon arrival, the temperature is measured. If a body temperature ≥ 38.0 °C is confirmed, immediate blood sampling with inflammation parameters and blood cultures is performed. Patients are then taken to the hospital room and placed in bed, where therapy with intravenous fluids and antibiotics is probably administered. Temperature is monitored in real time by skin-mounted temperature probes on the patient’s foot or hand and on the patients’ trunk in the axillary region where the temperature is closest to core temperature. Additionally, the temperature is recorded at least three times per day in both ears, documenting the higher measure. If and as long as the central-peripheral temperature difference is ≥ 0.5 °C, patients are gently warmed with hot water bottles. The goal of heat application is to have the patient’s extremities (especially the feet, which are usually relatively cool during the rising phase of a fever) reach a temperature of < 0.5 °C equal to that of the trunk. Heat application is performed by the nursing staff as directed by the attending oncologist and continues as long as the patient is comfortable. When the fever has reached the third phase of temperature drop, when the body attempts to lower temperature and patients usually begin to sweat, the application will be stopped. The heat applications can be performed throughout the inpatient stay as long as the patient tolerates them.

The second intervention is the avoidance of antipyretics, to enhance the effect of the first intervention. Parents and staff will be instructed not to use physical practices (e.g., cold compresses) or pharmacological measures (e.g., paracetamol, metamizol, or ibuprofen) with the intention to reduce body temperature in the first phase of fever, when the patient’s body increases the temperature to reach its new thermoregulatory set point. Any medically necessary exceptions should be documented and justified by the attending physician.

The control group will receive standard care and antipyretic treatment as usual. In the oncology center where the pilot study will be conducted, pharmacologic antipyretics are usually not administered, with exceptions justified by urgent need. In the final multicenter study, it must be assumed that there will be hospitals that practice more aggressive antipyretic treatment. At the first sign of fever or above a certain body temperature, they are likely to administer acetaminophen, metamizol, or ibuprofen to reduce the fever from the beginning. For evaluation, the central-peripheral body temperature difference is monitored as in the intervention group.

#### Criteria for discontinuing or modifying allocated interventions {11b}

Since no negative effects were reported in the underlying studies using heat application as an intervention in fever [32–38], we expect no harm. It is conceivable that both, chemotherapy and the underlying disease, may lead to dysregulation of the thermoregulatory control center, but this has not been reported so far. From this point of view, an upper limit should be introduced for the core body temperature above which measures should be initiated to reduce fever. A body temperature of ≥ 41.1 °C was chosen as the upper limit, as temperatures over 41 °C are remarkably rare [39, 40], and higher temperatures must be regarded as hyperpyrexia, an emergency case [41]. Therefore, when the core body temperature exceeds 41.0 °C, lowering of the body temperature by pharmacological antipyretic treatment with paracetamol, ibuprofen, or metamizol should be administered. However, since antipyretics are not effective or are unreliably effective if the thermoregulatory control center itself is disturbed, additional external cooling may have to be initiated. This decision must be examined individually in each case and underlies the responsibility of the attending physician.

Patients can be excluded from further participation within the study or withdraw themselves without having to provide any further reasons. Other possible reasons for dropout are expected to be:

- Withdrawal of the patient’s or legal representatives’ informed consent.
- An emerging condition that affects the efficacy of the study investigation or is contraindicated for the intervention.
- Retrospective assessment of either unmet inclusion criteria or met exclusion criteria as determined by the treating oncologist/lead investigator of the clinical trial.
- Medically necessary transfer of the patient to another department/hospital during the study phase.
- Unexpected findings that make continuation of therapy unjustifiable from an ethical or medical perspective. The decision is made by the principal investigator.

The whole study will be discontinued prematurely if it is perceptible, which relevant criteria cannot be fulfilled:

- The required recruitment number proves to be unachievable.
- The documentation is incomplete or intentionally filled out incorrectly.
- Legal or ethical requirements are not met.

#### Strategies to improve adherence to interventions {11c}

Interventions are administered by the nurses within the daily basic care. Adherence can be checked based on the daily documentation of the provided nursing measures or the medication from the patient’s record folder.

#### Relevant concomitant care permitted or prohibited during the trial {11d}

In the intervention group, antipyretics are only allowed to relieve pain or discomfort when other measures have been unsuccessful, but not with the primarily intent of lowering body temperature. Physical cooling measures are also not allowed. In the control group, any kind of heat application (e.g., increased room temperature) is not desired.

### Outcomes {12}

The objectives of this randomized pilot study are defined by the following outcomes:

1. Decision on study design (parallel vs. cross-over) based on inter- and intra-individual variability.
2. Assessment of patient, parent, and staff feedback is evaluated for optimizing the intervention.
3. Amount of adverse events is discussed for safety reasons.
4. The primary outcome is selected based on clinical significance and the greatest difference from treatment as usual. Further interesting variables will become the secondary outcome. The following potential outcome parameters will be tested:
  - Trajectory of central and peripheral body temperature.
  - Variability of approximation between central and peripheral body temperature.
  - Frequency and amount of medication (antipyretics/antibiotics) use on ward.
  - Clinical symptom progression including time to symptom freedom.
  - Physical well-being, assessed by a single-question survey for patients/caregivers.
  - Overall course of oncological disease and treatment.
5. Sample Size is calculated based on the effect size of the intervention.
6. Number of participants and obstacles encountered during recruitment are evaluated.
7. Reported covariates occurring during the study will be evaluated.
8. Required manpower and time are used for resource and cost calculation.

#### Sample size {14}

The hospital performing the randomized pilot study treats approximately 4–5 oncological patients with fever per month, so during half a year, about 24–30 cases may be included, depending on eligibility and dropout. This pilot study aims to include a total number of *n* = 24 cases with a 2:1 randomization, i.e., 16 in the intervention group and 8 in the control group. The larger sample size in the intervention group of this unequal randomization allows more variables to be tested and gives more power to detect adverse events of the intervention.

#### Recruitment and consent {15, 26a}

Every pediatric patient of pediatric oncology will be asked to participate in the study. The attending oncologist will make the patients aware of the ongoing study during the first consultation interview before starting chemotherapy. After this interview, patients or legal representatives have the option of agreeing to participate in the study. Informed consent is obtained by the attending oncologist via a standardized consent form. This procedure has been approved by the ethics committee. No study participants will be included who are in a direct relationship with, or dependent on, the research team (students, employees of the institution, close relatives). No further recruiting measures will be taken.

#### Participant timeline {13}

**Fig. 1:**
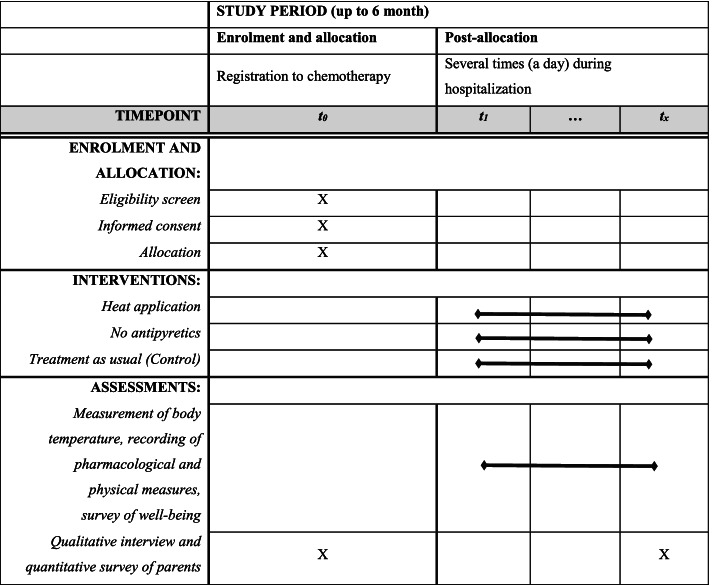
Schedule of enrollment, interventions, and assessments

When the patient is registered for treatment by chemotherapy, eligibility is tested, informed consent is obtained, and allocation is performed (*t*_0_). Intervention and assessments (measurement of body temperature, recording of pharmacological and physical measures, and survey of well-being) take place several times (a day) during hospitalization (*t*_1_− *t*_*x*_). Qualitative interview and quantitative survey of children’s parents are conducted before and after the patients’ hospitalization (*t*_*x*_). When the patient reappears in the hospital due to a new febrile episode, interventions and assessments start with *t*_1_. A schedule of the enrollment, interventions, and assessments is shown in Fig. 1.

### Assignment of interventions: allocation

#### Sequence generation {16a}

To ensure equality of structure, allocation will be made using an external service using a computer-generated, random allocation sequence on a 2:1 ratio.

#### Concealment mechanism {16b}

The department receives sequentially numbered, opaque, and sealed envelopes containing allocation and case report forms (CRF). These envelopes are assigned to each participating patient when registering for chemotherapy.

#### Implementation {16c}

The allocation sequence is generated by the study management. The participants are enrolled and assigned by the attending oncologist.

### Assignment of interventions: blinding

#### Who will be blinded {17a}

Blinding of participants, medical staff, and assessors is impossible, because all staff on the ward would realize if the patients receive any physical treatments. A sham intervention or placebo medication is not intended. Blinding of data analysts is also not intended for the pilot study.

#### Procedure for unblinding if needed {17b}

Not applicable, as there is no blinding.

### Data collection and management

#### Plans for assessment of outcomes and data management {18a, 19}

A majority of endpoints (body temperature, medications, number of febrile illnesses, mortality, etc.) are routinely entered into each patient’s standard medical record by hospital staff in both groups. Data will be transferred into the CRF by a researcher of the study team. The patients’ physical well-being during the febrile illness will be assessed by a single-question survey which is asked several times a day in the daily routine of the medical staff. Based on Sekhon et al.’s theoretical framework of acceptability [42] and its adaption for pilot studies [43], qualitative interviews and quantitative surveys with parents and clinical staff will be conducted. All parents of participating children will be asked to participate in a short qualitative interview and a survey after recruitment and after hospitalization. Parents will be asked questions about their perceptions and fears toward fever, fever management, and overall acceptability of the study. The clinic staff involved in the care of the patients will be interviewed and surveyed before, halfway through and after completion of the study. Questions for staff cover practicability of the intervention (heat application) in daily routine, assessment of temperature, assessment of well-being, and overall acceptance of the study. Data will be analyzed interpretive and iterative by the research team and obtained information is used for optimizing the main trial. Final data collection forms can be obtained from the author.

#### Plans to promote participant retention and complete follow-up {18b}

Follow-up is conducted regularly for all patients in the oncology department. The follow-up period is up to 6 months after the end of chemotherapy.

#### Additional consent provisions for collection and use of participant data and biological specimens {26b}

The data collected as part of the planned study will be collected pseudonymously, without mentioning names. The legal basis for the processing of personal data in clinical studies is voluntary written consent in accordance with the General Data Protection Regulation (EU-DSGVO) of April 27, 2016, and the amended Federal Data Protection Act (BDSG-neu) of May 25, 2018.

#### Confidentiality {27}

CRF will be stored in numerical order in a secure place and manner for a period of 10 years after completion of the study. All forms related to the study data will be kept in locked cabinets and access to the study data will be restricted.

#### Provisions for post-trial care {30}

To the extent that the hospital’s insurance does not regularly cover the study, subjects will be covered by additional insurance as part of the study.

#### Plans for collection, laboratory evaluation, and storage of biological specimens for genetic or molecular analysis in this trial/future use {33}

Not applicable, as laboratory evaluation and storage of biological specimens for genetic or molecular analysis is not part of the study.

## Statistical methods

### Statistical methods for primary and secondary outcomes {20a}

Statistical analysis of the study is performed at the Faculty of Health at Witten/Herdecke University. The analyses are primarily descriptive and exploratory and are used for study design and case number estimation and are performed under the supervision of a biometrician.

For the main study, all evaluations will be based on all randomized patients (intention-to-treat population), whether or not they adhered to the treatment protocol and whether or not they provided complete records. These are patients:

- Who discontinued the clinical trial; they are evaluated as if they had adhered to the trial.
- Who had therapy modified or changed; they will be analyzed in their original randomized group.
- Who took concomitant medications or received concomitant therapy without permission; they will continue to be included in the analysis.
- Whose planned investigations were not conducted in the scheduled time frame; they will continue to be included in the analysis.

Aside from the analysis of all randomized patients, a per-protocol analysis will also be carried out which is supposed to indicate which effect sizes can be reached under optimal circumstances. In the per-protocol population, all patients will be included who fulfill all study requirements. Patients who withdraw their consent to use their personal data for statistical analyses will be excluded from the analysis (dropouts).

### Interim analyses {21b}

Not applicable, as no interim analyses are planned for the pilot study.

### Methods for additional analyses (e.g., subgroup analyses) {20b}

Not applicable, as no additional analyses are planned at this time.

### Methods in analysis to handle protocol non-adherence and any statistical methods to handle missing data {20c}

Currently, missing values are not imputed. In the main study, missing values are replaced by a predictive regression model. It includes the final score as a dependent variable and baseline values, study group, and expectancy as independent variables.

### Plans to give access to the full protocol, participant-level data, and statistical code {31c}

Not applicable, as there are no plans to provide public access to the full protocol, participant-level data, and statistical code. A short version of the pilot and the main study will be published at drks.de.

### Oversight and monitoring

#### Composition of the coordinating center and trial steering committee {5d}

The study steering committee at Witten/Herdecke University will meet regularly during the course of the study. The responsible oncologist from the participating departments will participate via video conference. An external monitoring is not planned for the pilot study due to its exploratory nature.

#### Composition of the data monitoring committee, its role, and reporting structure {21a}

Not applicable, as due to the early stage of development and the absence of critical safety concerns, a separate data monitoring committee (DMC) is not required for this small, short-term pilot study.

#### Adverse event reporting and harms {22}

All adverse events (AE) and serious adverse events (SAE) observed during the study will be recorded. Each adverse event will be evaluated by the treating oncologist according to its severity and possible association with the experimental intervention under study. All adverse events will be evaluated according to the Common Terminology Criteria for Adverse Events (CTCAE) version 5.0 [44] and a decision made on whether to report them. If serious SAEs occur that seem to be related to the intervention, the principal investigator may terminate the study. Specifically, the trial will be terminated if adverse outcomes occur that call into question the safety of the experimental intervention.

#### Frequency and plans for auditing trial conduct {23}

The pilot study and compliance with the study protocol are currently not monitored. Monitoring is planned for the main study by the Center for Clinical Studies at Witten/Herdecke University (ZKS UW/H).

#### Plans for communicating important protocol amendments to relevant parties (e.g., trial participants, ethical committees) {25}

In case of necessary protocol amendments, all information will be submitted to the ethics committee and competent authority and implementation will be done after the approval.

#### Dissemination plans {31a}

The results and decisions of the pilot study will be incorporated into a dissertation and may be published. The study protocol of the main study as well as the final study results will also be published in peer-reviewed international journals.

## Discussion

Our main goal is to improve the care of children with cancer by providing the best possible support for febrile episodes. This randomized pilot study is intended to demonstrate the feasibility of heat application in febrile patients and to provide a basis for a main, multicenter, randomized trial in pediatric oncology. If fever can be supported in its progression by applying heat, this might reduce the thermoregulatory pressure and the mechanisms to increase core temperature, such as centralization, chills, and shivering. This would in turn decrease the discomfort of these symptoms. In addition, heat application could enable reaching optimal body temperature for immunological function faster and easier. Since all pediatric oncology patients with febrile neutropenia receive antibiotics immediately [45], these patients could perhaps additionally benefit from needing less stepping-up to second-line antibiotics with broader spectra, as some older studies suggested that antibiotics may be more effective at increased temperatures [46].

### Study limitations and bias

The limitation of this study is mainly founded in its pilot study design. The used sample size is small and not intended to show all clinical effects and aspects of heat application in fever. Since patients are aware of their group membership and there is no blinding, there is a risk of performance and detection bias. The results of this study should be used to plan the main trial, and therefore have no general validity. The oncological patients cannot be regarded as representative of other patient groups. Children, depending on their age, are not very good at formulating whether they are comfortable with the heat application or not. The patient population is very heterogeneous in terms of age and diseases. It must be assumed that each patient has its own risk profile and chance of survival. Therefore, we cannot determine the effect on prognosis.

## Trial status

The study is in final planning status to begin recruitment.

## Supplementary Information

**Additional file 1.** SPIRIT 2013 Checklist.

## Data Availability

Not applicable.

## Abbreviations

AE: Adverse event
CRF: Case report form
CTCAE: Common Terminology Criteria for Adverse Events
DMC: Data monitoring committee
RCT: Randomized controlled trial
SAE: Serious adverse event
